# Prevalence of hypertension and high-normal blood pressure among young adults in Zimbabwe: findings from a large, cross-sectional population-based survey

**DOI:** 10.1016/S2352-4642(23)00287-0

**Published:** 2023-12-06

**Authors:** Kalpana Sabapathy, Fredrick Cyprian Mwita, Ethel Dauya, Tsitsi Bandason, Victoria Simms, Chido Dziva Chikwari, Aoife M Doyle, David Ross, Anoop Shah, Richard J Hayes, Aletta E Schutte, Katharina Kranzer, Rashida Abbas Ferrand

**Affiliations:** Department of Infectious Disease Epidemiology and International Health London School of Hygiene & Tropical Medicine, London, UK; National Institute for Medical Research, Mwanza, Tanzania; The Health Research Unit Zimbabwe, Biomedical Research and Training Institute, Harare, Zimbabwe; The Health Research Unit Zimbabwe, Biomedical Research and Training Institute, Harare, Zimbabwe; Department of Infectious Disease Epidemiology and International Health London School of Hygiene & Tropical Medicine, London, UK; The Health Research Unit Zimbabwe, Biomedical Research and Training Institute, Harare, Zimbabwe; Department of Infectious Disease Epidemiology and International Health London School of Hygiene & Tropical Medicine, London, UK; The Health Research Unit Zimbabwe, Biomedical Research and Training Institute, Harare, Zimbabwe; Department of Infectious Disease Epidemiology and International Health London School of Hygiene & Tropical Medicine, London, UK; The Health Research Unit Zimbabwe, Biomedical Research and Training Institute, Harare, Zimbabwe; Institute for Life Course Health Research, Stellenbosch University, Tygerberg, South Africa; Department of Noncommunicable Disease Epidemiology, London School of Hygiene & Tropical Medicine, London, UK; Department of Infectious Disease Epidemiology and International Health London School of Hygiene & Tropical Medicine, London, UK; School of Population Health, University of New South Wales, Sydney, NSW, Australia; The George Institute for Global Health, Sydney, NSW, Australia; Department of Noncommunicable Disease Epidemiology and Department of Clinical Research, London School of Hygiene & Tropical Medicine, London, UK; The Health Research Unit Zimbabwe, Biomedical Research and Training Institute, Harare, Zimbabwe; Department of Noncommunicable Disease Epidemiology and Department of Clinical Research, London School of Hygiene & Tropical Medicine, London, UK; The Health Research Unit Zimbabwe, Biomedical Research and Training Institute, Harare, Zimbabwe

## Abstract

**Background:**

Hypertension is the greatest driver of cardiovascular mortality and onset might be in youth. We aimed to investigate the prevalence of and risk factors for elevated blood pressure (hypertension ≥140 mm Hg systolic, ≥90 mm Hg diastolic, or both) and high-normal blood pressure (130–139 mm Hg systolic, 85–89 mm Hg diastolic, or both) among youth in Zimbabwe.

**Methods:**

A population-based, cross-sectional survey of randomly sampled youth aged 18–24 years from 24 urban and peri-urban communities in three provinces (Harare, Bulawayo, and Mashonaland East) in Zimbabwe was conducted between Oct 4, 2021, and June 2, 2022. Standardised questionnaires were used by research assistants to collect sociodemographic, behavioural, and clinical data. Height, bodyweight, and blood pressure were recorded. Three seated blood pressure measurements were taken at standardised timepoints during participant interview using a digital sphygmomanometer and cuffs sized on mid-upper arm circumference. The association of potential risk factors with elevated blood pressure was examined using multivariable logistic regression.

**Findings:**

17 682 (94·4%) of 18 729 eligible participants were recruited, 17 637 (99·7%) of whom had complete data, and 16 883 (95·7%) of whom were included in the final study sample after excluding 754 (4·3%) pregnant women. The median age was 20 years (IQR 19–22), 9973 (59·1%) participants were female, and 6910 (40·9%) were male. The prevalence of hypertension was 7·4% (95% CI 7·0–7·8) and high-normal blood pressure was 12·2% (11·7–12·7). Overall, prevalence of hypertension was higher in men (8·7% [95% CI 8·2–9·6]) than in women (6·6% [6·0–6·9]), but with age increased to similar levels (at age 18 years 7·3% [6·2–8·6] and 4·3% [3·5–5·2]; at age 23–24 years 10·9% [9·3–12·6] and 9·5% [8·4–10·7] in men and women, respectively). After adjusting for factors associated with hypertension in the crude analysis, hypertension was associated with male sex (adjusted odds ratio 1·53 [95% CI 1·36–1·74]), increasing age (age 19–20 years 1·20 [1·00–1·44]; age 21–22 years 1·45 [1·20–1·75]; age 23–24 years 1·90 [1·57–2·30], *vs* age 18 years), and BMI of 30·0 kg/m^2^ or more (1·94 [1·53–2·47] *vs* 18·5–24·9 kg/m^2^). A BMI of 18·5 kg/m^2^ or less (0·79 [0·63–0·98] *vs* 18·5–24·9 kg/m^2^) and living with HIV (0·71 [0·55–0·92]) were associated with lower odds of hypertension.

**Interpretation:**

Prevalence of elevated blood pressure is high among urban and peri-urban youth in Zimbabwe and increases rapidly with age. Further research is needed to understand drivers of blood pressure elevation and the extent of target organ damage in youth in Zimbabwe and similar sub-Saharan African settings, to guide implementation of prevention and management strategies.

**Funding:**

Wellcome Trust.

## Introduction

Worldwide, 1·3 billion adults aged 30–79 years have hypertension, which is the commonest cause of cardiovascular disease.^1^ Raised systolic blood pressure is the leading cause of death globally, causing more than 10·8 million deaths (19% of all deaths) in 2019, and contributing to 9% of disability-adjusted life-years lost.^2^

In low-income and middle-income countries (LMICs) there has been a progressive increase in the number of adults with hypertension, surpassing that of high-income countries (HICs), with an estimated 1·04 billion people living with hypertension in LMICs compared with 349 million in HICs.^2,3^ Although hypertension was previously a condition associated with affluence, it is now one of poverty.^4^ Sub-Saharan Africa has been undergoing a rapid epidemiological transition, and the prevalence and incidence of hypertension are increasing.^5^ WHO estimates that Africa has the highest prevalence of hypertension and the highest age-adjusted rates of cardiovascular disease of any global region.^2^

Hypertension is usually understood to be a disease of advancing age. Existing international screening and treatment guidelines on hypertension are predominantly derived from studies in older adults with an average age of about 50 years and in people of European ancestry.^6–8^ Incidence of hypertension in individuals of African origin occurs earlier and blood pressure elevations are more severe than in those of European ancestry.^5^ Population surveys in sub-Saharan Africa among individuals with an average age of 30–50 years have shown a high prevalence of hypertension.^9–11^ However, evidence is accumulating that hypertension is prevalent even among adolescents and people younger than 30 years. A systematic review described the prevalence of hypertension among adolescents aged 10–19 years in sub-Saharan Africa, with prevalence ranging widely from less than 1% up to 25%.^12^ In studies including people aged 15–30 years in sub-Saharan Africa, 6–10% of participants have been reported to have hypertension.^9–11^ In one study, about a third of adolescents and young people had an elevated blood pressure, albeit below the threshold for hypertension.^13,14^ Elevated blood pressure frequently progresses to hypertension and is an independent risk factor for cardio-vascular disease, and elevations in youth track into adulthood.^15–17^ These studies have had small sample sizes, often recruited adolescents and young people as part of a larger group of adults and did not assess age-specific risk factors, or they have been conducted in schools in contexts where school attendance is not universal.^13,18^

There is scant understanding of the epidemiology of hypertension in young people, and especially so in sub-Saharan Africa.^5,19^ Zimbabwe is a land-locked country in southern Africa with a population of approximately 15 million people according to 2022 census data.^20^ The country is divided into ten provinces and the two largest cities are Harare and Bulawayo. Using data from a large population-representative survey in Zimbabwe, we aimed to investigate the population distribution of blood pressure and the prevalence of hypertension, including isolated systolic and diastolic hypertension, and high-normal blood pressure,^6^ and factors associated with hypertension among young adults in Zimbabwe.

## Methods

### Study design and participants

A population-based, cross-sectional survey was conducted in Zimbabwe among youth aged 18–24 years to ascertain the outcome of a cluster-randomised trial (CHIEDZA; NCT03719521) investigating the impact of community-based integrated HIV and sexual and reproductive health services for youth on population-level HIV outcomes. A prespecified objective of the survey was to investigate the prevalence of elevated blood pressure and associated factors.

The CHIEDZA trial protocol, including survey methods has been published.^21^ Briefly, the trial was conducted in 24 urban and peri-urban communities in three provinces (Harare, Bulawayo, and Mashonaland East), with eight communities per province. Zimbabwe has two main ethnic groups, Shona and Ndebele, and the study represents both ethnic groups. Bulawayo is predominantly Ndebele, and Mashonaland East and Harare are predominantly Shona. Each community had geographically demarcated areas that served as clusters. The 24 clusters were randomly assigned 1:1 to either standard of care (existing facility-based health services) or the intervention, stratified by province. Implementation of the trial outcome cross-sectional survey was staggered with recruitment in Harare from Oct 4 to Dec 15, 2021, in Bulawayo from Jan 4 to March 10, 2022, and in Mashonaland East from April 4, to June 2, 2022, aiming to recruit 16 800 participants (5600 per province).

Multistage sampling was used. Satellite images were used to map each building within a cluster onto OpenStreetMap, and ArcGIS was used to divide all streets within the cluster into short sections (approximately 100–200 m). A random sample of street sections was selected, and all residents of those sections were enumerated. All eligible individuals (aged 18–24 years) residing in households on either side of the selected section were approached for participation in the survey.

The study was approved by the Medical Research Council, Zimbabwe, the Biomedical Research and Training Institute Institutional Review Board and the ethics committee of the London School of Hygiene & Tropical Medicine. Participants viewed an information video about the study (in English, Shona, or Ndebele) on a tablet. Written informed consent was documented electronically on a tablet, with participants retaining a signed paper copy for their records. Participants with a systolic blood pressure equal to or greater than 180 mm Hg, a diastolic blood pressure equal to or greater than 100 mm Hg, or both, with or without symptoms, and a systolic blood pressure equal to or greater than 140 mm Hg, diastolic blood pressure equal to or greater than 90 mm Hg, or both, in the presence of consistent symptoms were referred to the nearest clinic for further management.

### Procedures

Trained research assistants carried out survey procedures according to standardised operating procedures.^21^ An interviewer-administered questionnaire was used to collect sociodemographic data, behavioural data, and medical history including pregnancy, knowledge of HIV status, history of chronic conditions, use of regular medication, and self-rated health. Sex was determined by self-report of biological sex at birth (female, male, or intersex). The International Physical Activity Questionnaire was used to ascertain levels of physical activity.^22^ The Alcohol Use Disorders Identification Test (AUDIT), a tenitem internationally validated tool, was used to screen for alcohol use disorder.^23^ The Shona Symptom Questionnaire (SSQ), a locally developed and validated 14-item scale, was used to screen for common mental health conditions.^24^

Bodyweight was measured to the nearest 0·1 kg using digital Seca 803 weight scales (Seca, Hamburg, Germany) in minimal clothing and with shoes removed. Height was measured to the nearest 0·1 cm using a Seca 213 stadiometer (Seca). Three seated blood pressure measurements were taken at standardised timepoints during the interview (at least 5 min apart) using a digital sphygmomanometer (Omron X2, Kyoto, Japan), with the first measure taken after 15 min of rest. Blood pressure measurements were performed using cuffs sized on mid-upper arm circumference (17–22 cm: small cuff; >22–32 cm: regular cuff; >32–42 cm: large cuff) according to WHO guidelines. A dried blood spot was collected for HIV antibody and viral load testing.

### Statistical analysis

The sample size of the survey was determined by the underlying assumptions and power needed for detecting a difference in primary outcome by group for the underlying trial for which the population-based survey was conducted.^21^

We excluded participants who reported they were pregnant, due to physiological differences in pregnancy that affect blood pressure and have the potential to distort BMI data. Age was examined in four categories (determined by the number of participants overall in each category, irrespective of outcome) to reflect approximately equivalent numbers of participants aged 18 years, 19–20 years, 21–22 years, and 23–24 years (ie, there were approximately equivalent numbers of participants aged 18 years as there were in each of the other categories). Self-reported household income was examined using standard categories, except the lowest income category was further subdivided to less than US$50 per month and $50–100 per month, rather than less than $100 per month, which is more commonly used, to capture extreme poverty in this setting.

Factor analysis of household assets was done to create a wealth index in quintiles based on self-report of household assets in working condition (namely, refrigerator, bicycle, car, television, radio, microwave, mobile phone, and computer or laptop). Levels of physical activity were expressed as multiples of the resting metabolic rate in minutes. A threshold of 8 on the AUDIT score (low risk <8 and high risk ≥8) was used to define alcohol use disorder or hazardous drinking.^23^ An SSQ score of 8 or more indicated a risk of common mental health conditions.^24^ Bodyweight was categorised according to BMI as follows: less than 18·5 kg/m^2^ (underweight); 18·5–24·9 kg/m^2^; 25·0–29·9 kg/m^2^ (overweight); and 30·0 kg/m^2^ or more (obesity). The mean of the second and third blood pressure readings was used to determine blood pressure outcomes.^7^ Blood pressure outcome categories were defined according to International Society of Hypertension guidelines as follows: hypertension as systolic blood pressure equal to or greater than 140 mm Hg, diastolic blood pressure equal to or greater than 90 mm Hg, or both; isolated systolic hypertension as systolic blood pressure equal to or greater than 140 mm Hg (with ≤89 mm Hg diastolic blood pressure); isolated diastolic hypertension as diastolic blood pressure equal to or greater than 90 mm Hg (with ≤139 mm Hg systolic blood pressure); and high-normal blood pressure as systolic 130–139 mm Hg, diastolic 85–89 mm Hg, or both.^6^

The prevalence and corresponding 95% CIs of hypertension and high-normal blood pressure by sex were calculated. The distributions of systolic and diastolic blood pressure in men and women were examined, and the prevalence of hypertension by age and BMI category were plotted in a two-way graph.

Logistic regression modelling was used to investigate factors associated with hypertension and high-normal blood pressure, adjusting a priori for cluster in the crude analysis to account for sampling strategy of the cluster-randomised trial for which the cross-sectional survey in this study was conducted. Factors associated with hypertension in the crude analysis at a significance level of p<0·05 were taken forward and included in separate multivariable models for each potential risk factor, to estimate the adjusted association of the factor with the outcome (hypertension and high-normal blood pressure). If colinearity between explanatory variables was plausible and considered likely based on previous knowledge and was supported by cross-tabulation of the data, only the variable that was more strongly associated with the outcome was retained in the models, and hence socioeconomic quintiles were chosen over household income. Variables with p<0·05 in the final models were regarded as independently associated with the outcome. Potential interactions between sex and age or between sex and BMI for the association with hypertension were examined. Data were analysed using Stata version 17.0.

### Role of the funding source

The funder of the study had no role in study design, data collection, data analysis, data interpretation, or writing of the report.

## Results

Of the 18729 eligible individuals identified by enumeration, 17 682 (94·4%) participants were contactable, consented and were recruited. Of these, 17637 (99·7%) participants had three blood pressure measures, and 754 (4·3%) pregnant women were excluded (appendix p 2). The final study sample included 16 883 (95·7%) participants with median age 20 years (IQR 19–22); 9973 (59·1%) participants were female and 6910 (40·9%) were male. More than half of participants had completed secondary education up to form 4 (ie, 10 years of schooling), with more than a quarter of all participants still in full-time education (table 1). Nearly half of all participants were neither in education nor employed (table 1).

Approximately one third of participants self-reported low levels of physical activity (table 1). High-risk alcohol use (AUDIT score ≥8) and ever smoking were both reported by less than 10% of participants (among women the prevalence of high-risk alcohol use and ever smoking was lower than among men). One-fifth of all participants were overweight (2675 [15·8%]) or had obesity (718 [4·3%]). Among women the prevalence of overweight and obesity was greater than among men (2015 [20·2%] and 629 [6·3%] *vs* 660 [9·6%] and 89 [1·3%]). Overall, less than a tenth of participants were underweight (783 [7·9%] among women and 818 [11·8%] among men; table 1).

Self-rated health was excellent or good in most individuals and only a small minority reported a previous diagnosis of diabetes, known renal disease, or hypertension (table 1). Of the 120 (0·7%) participants who were previously diagnosed with hypertension, 24 (20·0%) individuals were on treatment and 17 (14·2%) had a blood pressure of less than 140/90 mm Hg. 1166 (6·9%) participants were living with HIV, 391 (33·5%) of whom reported that they were taking antiretroviral therapy (table 1).

**Figure 1:**
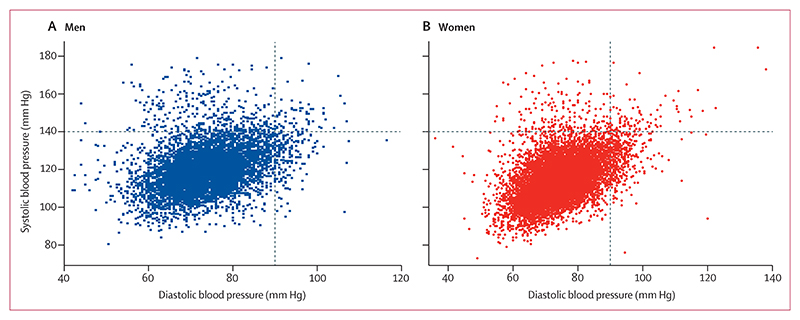
Scatter-plots of blood pressure (A) Blood pressure in men. (B) Blood pressure in women.

The first blood pressure measurement was slightly higher than the subsequent two measures (appendix p 6), supporting the use of the average of second and third measures to determine blood pressure. The population blood pressure was normally distributed with median systolic blood pressure of 116 mm Hg (IQR 110–124) and median diastolic blood pressure of 74 mm Hg (69–80; appendix p 3). The median systolic blood pressure was higher among men (119 mm Hg [IQR 112–127]) than women (114 mm Hg [108–122]). The median diastolic pressure was lower among men (73 mm Hg [69–79]) than women (75 mm Hg [70–80]). Isolated systolic hypertension was more frequent in men than women, whereas isolated diastolic hypertension was more frequent in women than men (figure 1).

**Figure 2:**
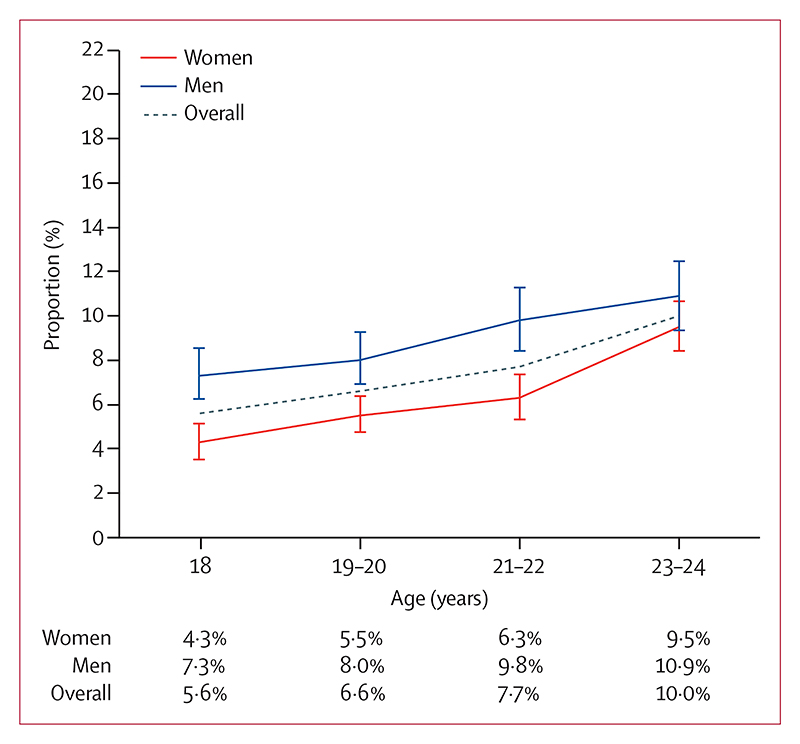
Prevalence of hypertension by age category in women and men

**Figure 3:**
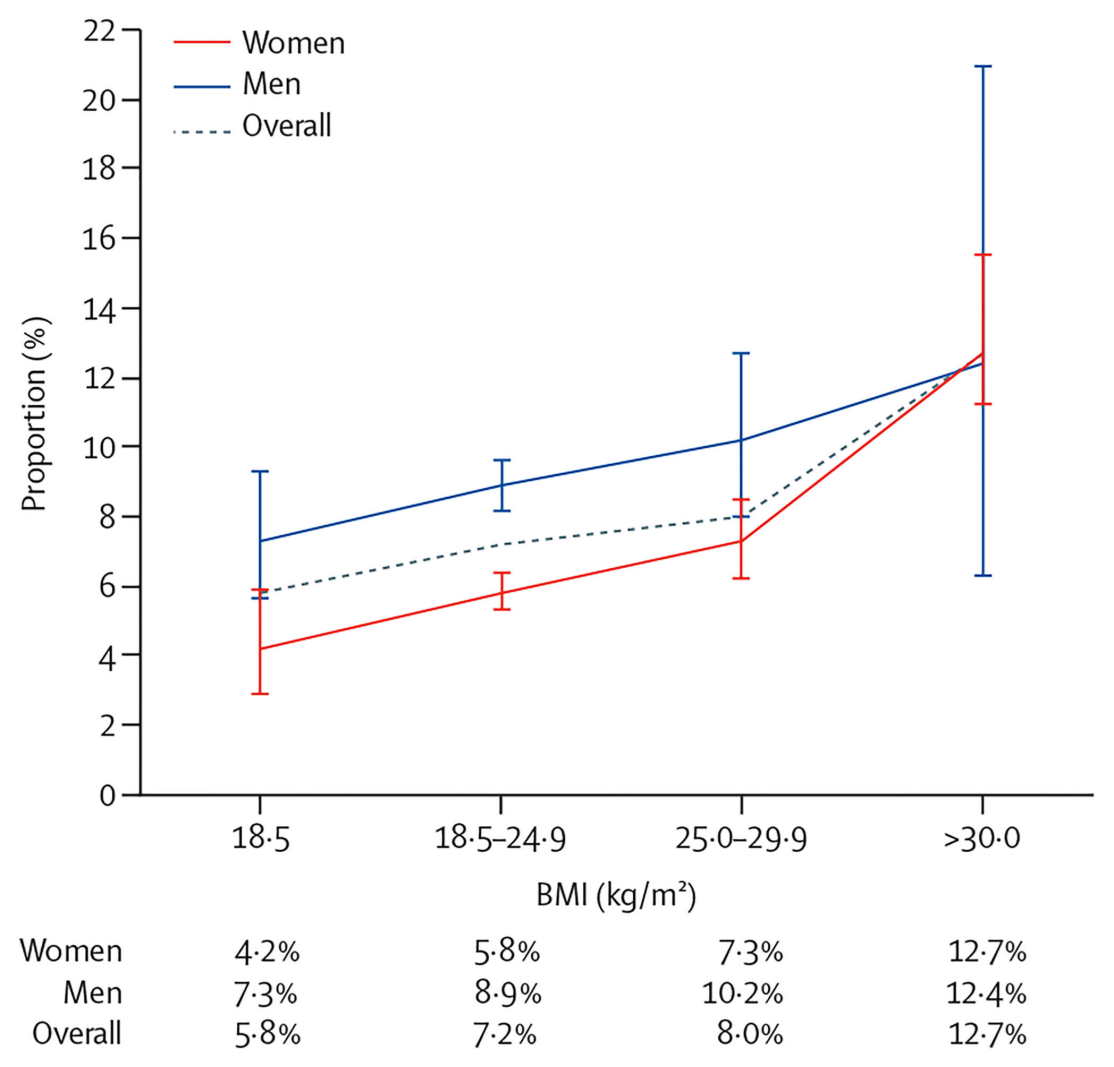
Prevalence of hypertension by BMI category in women and men

The prevalence of hypertension was 7·4% (95% CI 7·0–7·8), and high-normal blood pressure was 12·2% (11·7–12·7; table 2). The prevalence of isolated systolic hypertension was 3·2% (95% CI 2·9–3·5) and isolated diastolic hypertension was 3·4% (3·2–3·7; table 2). Hypertension was more prevalent among men (8·7% [8·2–9·6]) than women (6·6% [6·0–6·9]), which was driven by a higher prevalence of systolic blood pressure elevation in men (table 2 and figure 1). The rate of increase in prevalence of hypertension was greater for women than men, between the age categories 21–22 years and 23–24 years and the BMI categories overweight to obesity (figures 2, 3). Prevalence of hypertension was similar between the sexes by age 23–24 years (prevalence at age 18 years was 4·3% [95% CI 3·5–5·2] for women and 7·3% [6·2–8·6] for men, and prevalence at age 23–24 years was 9·5% [8·4–10·7] for women and 10·9% [9·3–12·6] for men). The effect of high BMI on systolic hypertension was especially marked among men (appendix p 4), but 6·3% (95% CI 5·8–6·8) of women had obesity compared with only 1·3% (1·0–1·6) of men. The prevalence of obesity was higher in women at all ages and increased sharply with age (appendix p 5).

Male sex, older age, higher level of education, occupation status, socioeconomic quintile, higher BMI, and HIV status were associated with having hypertension on crude analysis (table 3). After adjustment for these factors, being male remained strongly associated with hypertension (adjusted odds ratio [OR] 1·53 [95% CI 1·36–1·74); and obesity was the factor most strongly associated with hypertension (adjusted OR 1·94 [95% CI 1·53–2·47] compared with BMI 18·5–24·9 kg/m^2^). Age was also strongly associated with hypertension, with an increasing strength of association with increasing age (age 19–20 years adjusted OR 1·20 [95% CI 1·00–1·44], age 21–22 years 1·45 [1·20–1·75], and age 23–24 years 1·90 [1·57–2·30], compared with age 18 years; table 3). Being underweight (adjusted OR 0·79 [0·63–0·98]) and living with HIV (0·71 [0·55–0·92]) were associated with lower odds of hypertension (table 3). There was insufficient statistical evidence to support interactions between sex and age (p=0·093), or between sex and BMI (p=0·43) for the association with hypertension. The associations of higher level of education, occupation status, and socioeconomic quintile with hypertension was no longer apparent in multivariable models.

The same risk factors associated with hypertension were also associated with high-normal blood pressure after multivariable adjustment (appendix pp 7–8).

## Discussion

This large population survey from urban and peri-urban Zimbabwe, a sub-Saharan African country, presents blood pressure measures and risk factors for hypertension specifically among young adults, providing much needed robust data on blood pressure elevations in early adulthood. Our study found a high prevalence of hypertension despite the otherwise good health and young age of our population, with 7·4% of young adults aged 18–24 years (8·7% of men and 6·6% of women) already meeting criteria for hypertension (≥140 mm Hg systolic, ≥90 mm Hg diastolic, or both). A further 12·2% had high-normal blood pressure (130–139 mm Hg systolic, 85–89 mm Hg diastolic, or both) according to International Society of Hypertension guidelines, which use higher thresholds than current US guidelines (hypertension definition ≥130 mm Hg systolic, ≥80 mm Hg diastolic, or both).^25^ According to the US guidelines, 32% of our study sample would be classified as hypertensive.

Other data involving young adults in sub-Saharan Africa have shown similar prevalence of hypertension to that seen in our study from Zimbabwe. In Tanzania, the prevalence of hypertension was 6% in those aged 18–20 years and 9% in those aged 21–24 years.^13^ In Ethiopia, the prevalence was 10% for young people aged 18–25 years.^26^ Both studies involved around 600 young adults. Data from our study are consistent with these estimates, but the large scale and population random sampling provide greater confidence about the precision and representativeness of the estimates. The prevalence of hypertension in our study is higher than that from the UK as an example of a high-income country (4% in females and 7% in males aged 16–24 years), despite a much lower prevalence of obesity overall (4% in our study population compared with 12% in young people aged 18–24 years in the UK).^27,28^ Data from other high-income countries for comparison are scarce as prevalence is presented after age-standardisation and predominantly for adults older than 30 years, and data from the USA are based on a different threshold as already described.^2,25^ Among American adults, Black individuals had more than double the age-adjusted prevalence of hypertension than White individuals, Hispanic people, or Asian Americans.^29^

We observed a sharp rise in hypertension with age, from 5·6% at age 18 years to 10·0% by age 23–24 years. Reasons for the high prevalence and earlier onset of hypertension in sub-Saharan Africa include transitions from healthy traditional lifestyles (eg, diet and physical activity) to unhealthy alternatives, including consumption of processed foods high in salt, fat, and sugar, alcohol excess, lower physical activity, and exposure to air pollution.^19^ Lifetime exposure to sociodemographic pressure, which begins in utero, for instance with maternal malnutrition, might contribute to sympathetic activation and early vascular ageing; furthermore, a genetic predisposition common in sub-Saharan Africa causes abnormal sodium homeostasis and blood pressure regulation.^5^ A long-term US follow-up study found that by age 50 years, among individuals with onset of hypertension before age 35 years, 60% had single organ damage, and 25% had concurrent damage in more than one organ.^30^ Our data suggest that young adults in Zimbabwe might have harmful arterial pressures, which could accumulate with time with the potential to have substantial population impact. A systematic review that examined data on young adults with average follow-up of approximately 15 years, found the population attributable fraction for cardiovascular events associated with raised blood pressure (high-normal blood pressure and hypertension) was 24%.^31^ Although the data were from high-income and middle-income countries in Europe, North America, and Asia, and the age range of young adults in this analysis was 18–45 years at recruitment, the potential for similar impact in Zimbabwe and similar sub-Saharan African settings is alarming, especially given already existing challenges for public health systems.

Hypertension prevalence was higher among young men than young women, driven by systolic rather than diastolic elevation. The clinical relevance of isolated systolic hypertension in youth is debated. Growing evidence suggests that it is associated with excess cardiovascular risk at older age, and regular monitoring and lifestyle modification is advised.^32^ In both men and women, blood pressure increased from age 18 years to 23–24 years, with hypertension in women equivalent to that among men by age 24 years, which was probably explained by higher levels of obesity in women.

Although a relatively small proportion of the overall population had obesity, having a high BMI was associated with almost twice the odds of having hypertension, consistent with other studies across age groups.^2,14^ Crucially, the global prevalence of obesity has tripled over the past 50 years, and in sub-Saharan Africa, undernutrition and high BMI co-exist as public health challenges.^33^ In our study, a fifth of youth were overweight or had obesity, but a further 9% were underweight. In many cultures, higher bodyweight is traditionally perceived to be associated with better health and therefore nuanced, culturally sensitive messaging is required to address overnutrition and undernutrition. In our study, the prevalence of obesity increased from 2·4% to 7·1% between the ages of 18 years to 23–24 years, largely driven by the increase in prevalence among women. Being overweight was also highly prevalent among women, and this prevalence can be expected to increase with advancing age and for individuals with overweight to be more likely to have obesity with age. Consequently, the prevalence of hypertension in women as they age might even exceed that in men as seen in other studies in older Africans.^34^

Consistent with other studies from sub-Saharan Africa, people living with HIV were less likely to have hypertension even after adjusting for BMI.^35^ This contrasts with findings from HICs where HIV increases cardiometabolic risk. Possible explanations include ethnic differences and sociodemographic factors;^5^ however, further research is warranted. Although we did not find evidence to support an association between common mental health conditions and hypertension, further exploration of the effect of chronic stress on hypertension remains relevant.

A question that arises in studies that measure blood pressure at a single timepoint is whether these findings might represent an artificially elevated blood pressure as a result of so-called white-coat hypertension (blood pressure elevations that occur in the presence of health professionals), and whether a measure on a single occasion represents persistently elevated blood pressure. The clear age trends in our study, and association of hypertension with known risk factors consistent with other studies, suggest our findings are unlikely to be spurious. It is noteworthy that there was a higher proportion of women than men in the study, and that women were also more likely to have obesity, which would contribute to a higher prevalence of hypertension and high-normal blood pressure than in a population with equal proportions of men and women. However, only 629 (3·7%) of all participants were women with obesity. We also acknowledge that our data do not reflect rural Zimbabwean youth and findings should be interpreted with due consideration of the urban and peri-urban context and the higher proportion of women in the study communities. The high prevalence of high-normal blood pressure and the finding that risk factors for high-normal blood pressure were the same as for hypertension, illustrate that elevated blood pressure measures represent a continuum. Thus, our findings highlight an urgent and neglected public health problem in Zimbabwe and quite likely also for other similar settings in sub-Saharan Africa, with the consequent morbidity and mortality only likely to be visible in a decade. Intervening in this age group represents a timely opportunity to avert the considerable mortality and morbidity due to cardiovascular disease that is being observed among older adults, now the leading cause of death globally.

Further research to investigate blood pressure in youth in sub-Saharan Africa is urgently needed. Specifically, unattended blood pressure measures and 24 h ambulatory blood pressure monitoring would enable interrogation of both the possible white-coat effect and masked hypertension, a term that refers to the lack of normal lowering of blood pressure at night time (non-dipping).^6^ In addition, it is vital to interrogate the causal factors for development of hypertension. The advances in technologies to measure blood pressure, including non-invasive techniques for central blood pressure monitoring and organ imaging, and the advent of metabolomics, offer opportunities to interrogate whether these blood pressure measurements represent true hypertension associated with metabolic perturbations, and whether there is end-organ damage, well before cardiovascular events occur.^36,37^ The optimal timing to recommend drug treatment is a further complex context-specific question that should be examined and weighed up, considering the realities of health systems in sub-Saharan Africa against benefits to individual health.

A key strength of this study was that it had a large sample size, with a population-representative sample of urban and peri-urban youth. The survey was conducted across a large number of peri-urban and urban communities across the country and participation rates were high. There was a higher proportion of women than men in the survey. However, this was not explained by differential participation rates. Among those enumerated age 18–25 years in all three provinces, approximately 60% were women and 40% were men. Further enquiry suggested that the sex imbalance was due to migration of young men away from home for economic reasons. Multiple risk behaviours and health outcomes were assessed, and validated data collection tools and international guidelines for blood pressure measurements and definitions were used. A limitation of our study was that blood pressure was measured during a single visit. However, other internationally accepted standards were adhered to, with three seated measurements taken at standardised intervals and the average of the second and third measures used to determine hypertension, an approach supported by our finding that the first measure was consistently higher than the latter two measures.^7^ Assessment of other traditional risk factors for cardiovascular disease such as dyslipidaemia and hyperglycaemia was not undertaken. Behaviour data (eg, alcohol consumption or physical activity) were self-reported and might be subject to recall or desirability bias but it is unlikely that it was differential by blood pressure measure.

In summary, our study demonstrates that hypertension is already prevalent at an alarming level among youth in urban and peri-urban settings in Zimbabwe. Further research to understand the drivers of hypertension, impact on target organs, as well as sustainable lifestyle modification strategies and indications for treatment initiation in youth in sub-Saharan Africa, is urgently needed.

## Supplementary Material

Supplementary Material

## Figures and Tables

**Table 1:** Participant characteristics

	Population overall (n=16 883)	Women (n=9973)	Men (n=6910)
**Sociodemographic characteristics**			
Age category, years			
18	4097 (24·3%)	2307 (23·1%)	1790 (25·9%)
19–20	4818 (28·5%)	2767 (27·7%)	2051 (29·7%)
21–22	3927 (23·3%)	2303 (23·1%)	1624 (23·5%)
23–24	4041 (23·9%)	2596 (26.0%)	1445 (20.9%)
Highest education attained			
Primary or below	3062 (18·1%)	2128 (21·3%)	934 (13·5%)
Secondary form 4	10 271 (60·8%)	6026 (60.4%)	4245 (61.4%)
Secondary form 6	2179 (12·9%)	1086 (10·9%)	1093 (15·8%)
Higher education	1371 (8·1%)	733 (7·3%)	638 (9·2%)
Occupation			
In education	4898 (29·0%)	2518 (25·2%)	2380 (34·4%)
Employed or own business	802 (4·8%)	347 (3·5%)	455 (6·6%)
Work in informal sector	3051 (18·1%)	1534 (15·4%)	1517 (22·0%)
None of the above	8132 (48·2%)	5574 (55·9%)	2558 (37·0%)
Household income per month, US$			
Less than 50	2506 (14·8%)	1531 (15·4%)	975 (14·1%)
50–100	4241 (25·1%)	2548 (25·5%)	1693 (24·5%)
101–200	4515 (26.7%)	2637 (26·4%)	1878 (27·2%)
201–500	2595 (15·4%)	1449 (14·5%)	1146 (16·6%)
More than 500	583 (3·5%)	289 (2·9%)	294 (4·3%)
Do not know or missing	2443 (14·5%)	1519 (15·2%)	924 (13·4%)
Socioeconomic quintile			
Lowest quintile (least affluent)	3727 (22.1%)	2465 (24·7%)	1262 (18·3%)
Second lowest quintile	2966 (17·6%)	1846 (18·5%)	1120 (16·2%)
Middle quintile	3401 (20·1%)	1975 (19·8%)	1426 (20.6%)
Second highest quintile	3349 (19·8%)	1887 (18·9%)	1462 (21·2%)
Highest quintile (most affluent)	3440 (20.4%)	1800 (18·0%)	1640 (23·7%)
**Behavioural characteristics**			
Physical activity, resting metabolic rate^*^			
Low	5983 (35·4%)	4170 (41·8%)	1813 (26·2%)
Moderate	5523 (32·7%)	3265 (32·7%)	2258 (32.7%)
High	5163 (30·6%)	2433 (24·4%)	2730 (39·5%)
Do not know or missing	214 (1·3%)	105 (1·1%)	109 (1·6%)
Alcohol use or risk of problem drinking			
Never drink or low-risk alcohol use	15 442 (91·5%)	9536 (95·6%)	5906 (85·5%)
Increased risk—possible dependence, AUDIT score ≥8†	1426 (8·4%)	432 (4·3%)	994 (14·4%)
Do not know or missing	15 (0·1%)	5 (0·1%)	10 (0·1%)
Smoking history			
Never smoker	15 819 (93·7%)	9864 (98·9%)	5955 (86.2%)
Ever smoker	1053 (6·2%)	105 (1·1%)	948 (13·7%)
Do not know or missing	11 (0·1%)	4 (<0·1%)	7 (0·1%)
**Clinical characteristics**			
Self-rated health in previous 12 months			
Excellent	6961 (41·2%)	3802 (38·1%)	3159 (45·7%)
Good	8562 (50·7%)	5245 (52·6%)	3317 (48·0%)
Fair	1252 (7·4%)	839 (8·4%)	413 (6·0%)
Poor	108 (0·6%)	87 (0·9%)	21 (0·3%)
Known hypertension			
No	16 762 (99·3%)	9867 (98·9%)	6895 (99·8%)
Yes	120 (0.7%)	105 (1·1%)	15 (0.2%)
Do not want to say	1 (<0·1%)	1 (<0·1%)	0
Known diabetes			
No	16 857 (99·9%)	9960 (99·9%)	6897 (99·8%)
Yes	24 (0.1%)	11 (0.1%)	13 (0.2%)
Do not want to say	2 (<0·1%)	2 (<0·1%)	0
Known renal disease			
No	16 864 (99·9%)	9962 (99·9%)	6902 (99·9%)
Yes	17 (0.1%)	10 (0.1%)	7 (0.1%)
Do not want to say	2 (<0·1%)	1 (<0·1%)	1 (<0·1%)
SSQ			
Low risk of common mental health condition	15 737 (93·2%)	9105 (91·3%)	6632 (96·0%)
Risk of common mental health condition, SSQ score ≥8‡	1146 (6.8%)	868 (8.7%)	278 (4·0%)
BMI category, kg/m^2^			
Less than 18·5	1601 (9·5%)	783 (7·9%)	818 (11.8%)
18·5–24·9	11 888 (70·4%)	6545 (65·6%)	5343 (77·3%)
25·0–29·9	2675 (15·8%)	2015 (20·2%)	660 (9·6%)
30·0 or more	718 (4·3%)	629 (6·3%)	89 (1·3%)
Missing	1 (<0·1%)	1 (<0·1%)	0
HIV status			
Not known to be living with HIV	15 599 (92·4%)	9106 (91·3%)	6493 (94·0%)
Known to be living with HIV	1166 (6.9%)	802 (8·0%)	364 (5·3%)
Missing or confirmatory results unavailable	118 (0·7%)	65 (0·7%)	53 (0·8%)

AUDIT=Alcohol Use Disorders Identification Test. SSQ=Shona Symptom Questionnaire.

* The International Physical Activity Question-naire was used to ascertain levels of physical activity, expressed as metabolic equivalent of task minutes, based on the The International Physical Activity Questionnaire protocol, which defines physical activity according to duration and intensity, then categorised into three levels of low, medium, and high.

† Threshold of 8 on the AUDIT score: low risk <8 and high risk ≥8.

‡ Standard SSQ thresholds of ≥8 to indicate risk of common mental health conditions was used versus <8 for low risk.

**Table 2:** Prevalence of hypertension, systolic and diastolic hypertension, and high-normal blood pressure

	Population overall (n=16 883)	Women (n=9973)	Men (n=6910)
Hypertension: ≥140 mm Hg systolic, ≥90 mm Hg diastolic, or both	1254, 7·4% (7·0–7·8)	642, 6·6% (6·0–6·9)	612, 8·7% (8·2–9·6)
Systolic hypertension: ≥140 mm Hg with any diastolic	698, 4·1% (3·8–4·4)	290, 2·8% (2·5–3·1)	422, 6·1% (5·6–6·7)
Isolated systolic hypertension: ≥140/≤89 mm Hg	542, 3·2% (2·9–3·6)	187, 1·9% (1·6–2·2)	355, 5·1% (4·6–5·7)
Diastolic hypertension: ≥90 mm Hg with any systolic	712, 4·3% (4·0–4·6)	455, 4·6% (4·2–5·0)	257, 3·7% (3·3–4·2)
Isolated diastolic hypertension: ≤139/≥90 mm Hg	556, 3·4% (3·2–3·7)	366, 3·7% (3·3–4·1)	190, 2·7% (2·4–3·2)
High-normal blood pressure: 130–139 mm Hg systolic, 85–89 mm Hg diastolic, or both	2064, 12·2% (11·7–12·7)	1033, 10·4% (9·8–11·0)	1031, 14·9% (14·1–15·8)

Data are n, % (95% CI).

**Table 3:** Factors associated with hypertension

	Hypertension: ≥140 mm Hg systolic, ≥90 mm Hg diastolic, or both (n=1254)	Crude OR^*^(95% CI†)	p value for crude OR	Multivariable OR‡ (95% CI^†^)	p value for multivariable OR
Sex	··	··	<0·0001	··	<0·0001
Female	642 (6·4%)	1 (ref)	··	1 (ref)	··
Male	612 (8·9%)	1·46 (1·30–1·64)	··	1·53 (1·36–1·74)	··
Age category, years	··	··	<0·0001	··	<0·0001
18	229 (5·6%)	1 (ref)	··	1 (ref)	··
19–20	317 (6·6%)	1·21 (1·02–1·44)	··	1·20 (1·00–1·44)	
21–22	304 (8·0%)	1·46 (1·22–1·74)	··	1·45 (1·20–1·75)	··
23–24	404 (10·0%)	1·88 (1·59–2·23)	··	1·90 (1·57–2·30)	··
Highest education attained	··	··	<0·0001	··	073
Primary or below	212 (6·9%)	1 (ref)	··	1 (ref)	··
Secondary form 4	744 (7·2%)	1·12 (0·95–1·31)	··	1·00 (0·85–1·18)	··
Secondary form 6	164 (7·5%)	1·29 (1·03–1·58)	··	0·90 (0·71–1·14)	··
Higher education (above form 6)	134 (9·8%)	1·61 (1·28–2·02)	··	1·04 (0·80–1·35)	··
Occupation	··	··	0·0039	··	036
In education	365 (7·5%)	1 (ref)	··	1 (ref)	··
Employed or own business	75 (9·4%)	1·27 (0·98–1·65)	··	0·97 (0·74–1·27)	··
Work in informal sector	247 (8·1%)	1·07 (0·90–1·27)	··	0·88 (0·72–1·06)	··
None of the above or unemployed	567 (7·0%)	0·87 (0·76–1·00)	··	0·87 (0·74–1·02)	··
Socioeconomic quintile	··	··	0·042	··	0·61
Lowest quintile (least affluent)	285 (7·6%)	1 (ref)	··	1 (ref)	··
Second lowest quintile	214 (7·2%)	1·04 (0·86–1·26)	··	0·92 (0·76–1·11)	··
Middle quintile	242 (7·1%)	1·06 (0·88–1·28)	··	0·89 (0·75–1·07)	··
Second highest quintile	233 (7·0%)	1·06 (0·88–1·29)	··	0·86 (0·71–1·03)	··
Highest quintile (most affluent)^3^	280 (8·1%)	1·30 (1·08–1·57)	··	0·97 (0·81–1·17)	··
Physical activity, resting metabolic rate§	··	··	0·85	··	043
Low	444 (7·4%)	1 (ref)	··	1 (ref)	··
Moderate	388 (7·0%)	0·99 (0·82–1·11)	··	0·89 (0·77–1·03)	··
High	371 (7·2%)	0·98 (0·84–1·13)	··	0·85 (0·73–0·99)	··
Do not know or missing	51 (23·8%)	NA	··	NA	··
Alcohol use or risk of problem drinking	··	··	0·061	··	1O0
Never drink or low-risk alcohol use	1182 (7·4%)	1 (ref)	··	1 (ref)	··
Increased risk—possible dependence, AUDIT score ≥8¶	70 (8·7%)	1·29 (1·00–1·66)	··	1·00 (0·76–1·30)	··
Do not know or missing	2 (13·3%)	NA	··	NA	··
Smoking history	··	··	0·22	··	0·46
Never smoker	1162 (7·4%)	1 (ref)	··	1 (ref)	··
Ever smoker	89 (8·5%)	1·16 (0·92–1·45)	··	0·93 (0·74–1·18)	··
Do not know or missing	3 (27·2%)	NA	··	NA	··
BMI category, kg/m^2^	··	··	<0·0001	··	<00001
Less than 18·5	93 (5·8%)	0·79 (0·64–0·99)	··	0·79 (0·63–0·98)	··
18·5–24·9	856 (7·2%)	1 (ref)	··	1 (ref)	··
25·0–29·9	214 (8·0%)	1·13 (0·97–1·33)	··	1·17 (1·00–1·38)	··
30·0 or more	91 (12·7%)	1·92 (1·52–2·42)	··	1·94 (1·53–2·47)	··
SSQ	··	··	0·29	··	0·39
Low risk of common mental disorder	1176 (7·5%)	1 (ref)	··	1 (ref)	··
Risk of common mental disorder, SSQ score ≥8‖	78 (6·8%)	0·88 (0·69–1·12)	··	0·93 (0·73–1·18)	··
HIV status	··	··	0·022	··	0·023
Living without HIV	1117 (7·6%)	1 (ref)	··	1 (ref)	··
Living with HIV	65 (5·6%)	0·74 (0·57–0·96)	··	0·71 (0·55–0·92)	··
Missing or confirmatory results unavailable	72 (61·0%)	NA	··	NA	··

AUDIT=Alcohol Use Disorders Identification Test. NA=not applicable. SSQ=Shona Symptom Questionnaire.

* Crude OR adjusted a priori for cluster.

† Calculated using the likelihood ratio test.

‡ Separate models for each risk factor adjusted for sex, age, socioeconomic quintile, occupation, BMI, and HIV status.

§ The International Physical Activity Questionnaire was used to ascertain levels of physical activity, expressed as metabolic equivalent of task minutes, based on the The International Physical Activity Questionnaire protocol, which defines physical activity according to duration and intensity, then categorised into three levels of low, medium, and high. ¶Threshold of 8 on the AUDIT score: low risk <8 and high risk ≥8. ||Standard SSQ thresholds of ≥8 to indicate risk of common mental disorders was used versus <8 for low risk.

## Data Availability

Individual, anonymised participant data and a data dictionary will be available through the London School of hygiene & Tropical Medicine repository (Data Compass) 12 months after publication of trial results. Data will be available to anyone for further analyses with approval from the Medical Research Council of Zimbabwe.

## Research in context

### Evidence before this study

We searched PubMed for English language articles published between Jan 1, 2003, and Sept 1, 2023, using the MESH search terms “hypertension”, “youth or young people or adolescents”, and “Africa”. A meta-analysis of studies of hypertension in Africa of children and adolescents aged 3–19 years conducted between 2017 and 2020 reported a pooled prevalence of hypertension of 7·5% (defined as systolic or diastolic blood pressure ≥95th centile) and a prevalence of elevated blood pressure of 11·4% (systolic or diastolic blood pressure between 90th and 95th centile). The prevalence of hypertension in the studies included ranged from 0·2% to 39%. Another meta-analysis of 36 studies in Africa of adolescents aged 10–19 years reported a pooled prevalence of elevated blood pressure of 9·9%. The prevalence ranged from 0·2% to 25%. Some studies were considered in both meta-analyses and studies using both manual and digital automated measures were included. The definitions of hypertension and elevated blood pressure were variable, and some studies used centile-based criteria as defined above for paediatric cohorts (children aged <13 years), or threshold-based definitions, which in turn were inconsistent, with some using US guideline definitions of hypertension (blood pressure ≥130/≥80 mm Hg) rather than the threshold in all other international guidelines (blood pressure ≥140/≥90 mm Hg). Several studies did not define the setting from where participants were recruited and many involved individuals from schools, despite the fact that school attendees might not be representative of children and adolescents in the general population for socioeconomic reasons. Studies had relatively small samples and did not systematically investigate risk factors for hypertension. In population studies, age ranges have been highly variable and data poorly disaggregated by age; for example, many studies present data on young people aged 15–29 years as a single age category. Yet it is recognised that blood pressure tracks with age and elevated blood pressures might progress to hypertension with age. Therefore age-specific data are needed

### Added value of this study

Our study of nearly 17 000 youth aged 18–24 years found a high prevalence of both hypertension and high-normal blood pressure (sometimes referred to as prehypertension and defined as elevated blood pressure [above normal] but not meeting the criteria for hypertension) in urban and peri-urban settings in Zimbabwe. We found a marked increase in prevalence with age across a 7-year age-span and a higher prevalence among men than women. Consistent with other studies in both adults and children, a BMI of 30·0 kg/m^2^ or more was associated with increased odds of hypertension. Hypertension and high-normal blood pressure showed the same variations with age and sex and associations with BMI, suggesting that the phenomena represent a continuum. The large sample size, the population survey design with random sampling, and the use of internationally accepted methods to ascertain blood pressure elevations help provide confidence in the validity and precision of the estimates obtained.

### Implications of all the available evidence

Existing international screening and treatment guidelines are aimed at older adults, derived predominantly from studies on those older than 30 years from high-income settings. These guidelines ignore the growing body of evidence that suggests that onset of hypertension occurs much earlier in the life-course and is already prevalent in youth, and indeed increases with age even in young adulthood. Our study raises important questions that need further research, namely the drivers and natural history of hypertension, and the effect of blood pressure elevations on target organs in youth. This age group presents a crucial window of opportunity to prevent the considerable morbidity and mortality associated with cardiovascular disease, the leading cause of death globally. Whether or not screening should be instituted, how to promote sustainable healthy lifestyle behaviours among young people, whether treatment regimens are likely to have a long-term beneficial effect and, if so, what treatment regimens should be used, are all questions that need to be urgently addressed.

## Box

For **OpenStreetMap** see https://openstreetmap.us/

For **ArcGIS** see https://www.arcgis.com/

For **Data Compass** see https://datacompass.lshtm.ac.uk/

## References

[1] WHO Hypertension.

[2] Zhou B, Carrillo-Larco RM, Danaei G (2021). Worldwide trends in hypertension prevalence and progress in treatment and control from 1990 to 2019: a pooled analysis of 1201 population-representative studies with 104 million participants. Lancet.

[3] Mills KT, Bundy JD, Kelly TN (2016). Global disparities of hypertension prevalence and control: a systematic analysis of population-based studies from 90 countries. Circulation.

[4] Zhou B, Perel P, Mensah GA, Ezzati M (2021). Global epidemiology, health burden and effective interventions for elevated blood pressure and hypertension. Nat Rev Cardiol.

[5] Schutte AE, Botha S, Fourie CMT (2017). Recent advances in understanding hypertension development in sub-Saharan Africa. J Hum Hypertens.

[6] Unger T, Borghi C, Charchar F (2020). 2020 International Society of Hypertension Global Hypertension practice guidelines. Hypertension.

[7] WHO (2018). HEARTS technical package for cardiovascular disease management in primary health care: evidence-based treatment protocols.

[8] WHO (2021). Guideline for the pharmacological treatment of hypertension in adults: summary.

[9] Price AJ, Crampin AC, Amberbir A (2018). Prevalence of obesity, hypertension, and diabetes, and cascade of care in sub-Saharan Africa: a cross-sectional, population-based study in rural and urban Malawi. Lancet Diabetes Endocrinol.

[10] Yaya S, Ekholuenetale M, Bishwajit G (2018). Differentials in prevalence and correlates of metabolic risk factors of non-communicable diseases among women in sub-Saharan Africa: evidence from 33 countries. BMC Public Health.

[11] Beaney T, Schutte AE, Tomaszewski M (2018). May measurement month 2017: an analysis of blood pressure screening results worldwide. Lancet Glob Health.

[12] Chen A, Waite L, Mocumbi AO (2023). Elevated blood pressure among adolescents in sub-Saharan Africa: a systematic review and meta-analysis. Lancet Glob Health.

[13] Nsanya MK, Kavishe BB, Katende D (2019). Prevalence of high blood pressure and associated factors among adolescents and young people in Tanzania and Uganda. J Clin Hypertens (Greenwich).

[14] Crouch SH, Soepnel LM, Kolkenbeck-Ruh A (2021). Paediatric hypertension in Africa: a systematic review and meta-analysis. EClinicalMedicine.

[15] Gartlehner G, Vander Schaaf EB, Orr C, Kennedy SM, Clark R, Viswanathan M (2020). Screening for hypertension in children and adolescents: updated evidence report and systematic review for the US Preventive Services Task Force. JAMA.

[16] Guo X, Zou L, Zhang X (2011). Prehypertension: a meta-analysis of the epidemiology, risk factors, and predictors of progression. Tex Heart Inst J.

[17] Chen X, Wang Y (2008). Tracking of blood pressure from childhood to adulthood: a systematic review and meta-regression analysis. Circulation.

[18] Afrifa-Anane E, Agyemang C, Codjoe SN, Ogedegbe G, de-Graft Aikins A (2015). The association of physical activity, body mass index and the blood pressure levels among urban poor youth in Accra, Ghana. BMC Public Health.

[19] Olsen MH, Angell SY, Asma S (2016). A call to action and a lifecourse strategy to address the global burden of raised blood pressure on current and future generations: the Lancet Commission on hypertension. Lancet.

[20] Zimbabwe Data Portal (2022). Zimbabwe 2022 population and housing census.

[21] Dziva Chikwari C, Dauya E, Bandason T (2022). The impact of community-based integrated HIV and sexual and reproductive health services for youth on population-level HIV viral load and sexually transmitted infections in Zimbabwe: protocol for the CHIEDZA cluster-randomised trial. Wellcome Open Res.

[22] Craig CL, Marshall AL, Sjöström M (2003). International physical activity questionnaire: 12-country reliability and validity. Med Sci Sports Exerc.

[23] WHO (2001). AUDIT: the Alcohol Use Disorders Identification Test: guidelines for use in primary health care.

[24] Patel V, Simunyu E, Gwanzura F, Lewis G, Mann A (1997). The Shona Symptom Questionnaire: the development of an indigenous measure of common mental disorders in Harare. Acta Psychiatr Scand.

[25] Whelton PK, Carey RM, Aronow WS (2018). 2017 ACC/AHA/AAPA/ABC/ACPM/AGS/APhA/ASH/ASPC/NMA/PCNA guideline for the prevention, detection, evaluation, and management of high blood pressure in adults: a report of the American College of Cardiology/American Heart Association Task Force on Clinical Practice Guidelines. Hypertension.

[26] Demisse AG, Greffie ES, Abebe SM (2017). High burden of hypertension across the age groups among residents of Gondar city in Ethiopia: a population based cross sectional study. BMC Public Health.

[27] UK Goverment Office for Health Improvement and Disparities (2022). Obesity profile: short statistical commentary.

[28] Office for National Statistics UK Risk factors for undiagnosed high blood pressure in England: 2015 to 2019.

[29] Aggarwal R, Chiu N, Wadhera RK (2021). Racial/ethnic disparities in hypertension prevalence, awareness, treatment, and control in the United States, 2013 to 2018. Hypertension.

[30] Suvila K, McCabe EL, Lehtonen A (2019). Early onset hypertension is associated with hypertensive end-organ damage already by midlife. Hypertension.

[31] Luo D, Cheng Y, Zhang H (2020). Association between high blood pressure and long term cardiovascular events in young adults: systematic review and meta-analysis. BMJ.

[32] Yano Y, Lloyd-Jones DM (2016). Isolated systolic hypertension in young and middle-aged adults. Curr Hypertens Rep.

[33] WHO (2021). Obesity and overweight: key facts.

[34] Stewart S, Libhaber E, Carrington M (2011). The clinical consequences and challenges of hypertension in urban-dwelling black Africans: insights from the Heart of Soweto Study. Int J Cardiol.

[35] Dillon DG, Gurdasani D, Riha J (2013). Association of HIV and ART with cardiometabolic traits in sub-Saharan Africa: a systematic review and meta-analysis. Int J Epidemiol.

[36] Kollias A, Lagou S, Zeniodi ME, Boubouchairopoulou N, Stergiou GS (2016). Association of central versus brachial blood pressure with target-organ damage: systematic review and meta-analysis. Hypertension.

[37] Onuh JO, Aliani M (2020). Metabolomics profiling in hypertension and blood pressure regulation: a review. Clin Hypertens.

