# Supplementary material for: Prevalence of hypertension and high-normal blood pressure among young adults in Zimbabwe: findings from a large, cross-sectional population-based survey
Source: Lancet Child Adolesc Health. Author manuscript; Available in PMC 2025 Jul 17. (PMC7617873; doi:10.1016/S2352-4642(23)00287-0)
Supplement: Supplementary Material [file EMS206795-supplement-Supplementary_Material.pdf]

THE LANCET
Child & Adolescent Health

Supplementary appendix

This appendix formed part of the original submission and has been peer reviewed. We post it as supplied by the authors.

Supplement to: Sabapathy K, Mwita FC, Dauya E. Prevalence of hypertension and high-normal blood pressure among young adults in Zimbabwe: findings from a large, cross-sectional population-based survey. *Lancet Child Adolesc Health* 2023; published online Dec 6. https://doi.org/10.1016/S2352-4642(23)00287-0.

Contents

Supplementary Figure 1: Flow-chart of study participants 2
Supplementary information on data management: 3
Supplementary Figure 2: Distribution of population systolic and diastolic BP 3
Supplementary Figure 3: Systolic hypertension (irrespective of diastolic BP) by BMI category .. 4
Supplementary Figure 4: Prevalence of obesity with age 5
Supplementary Table 1: Median BP measures at first, second and third reading 6
Supplementary Table 2: Factors associated with high-normal BP 7

Supplementary Figure 1: Flow-chart of study participants

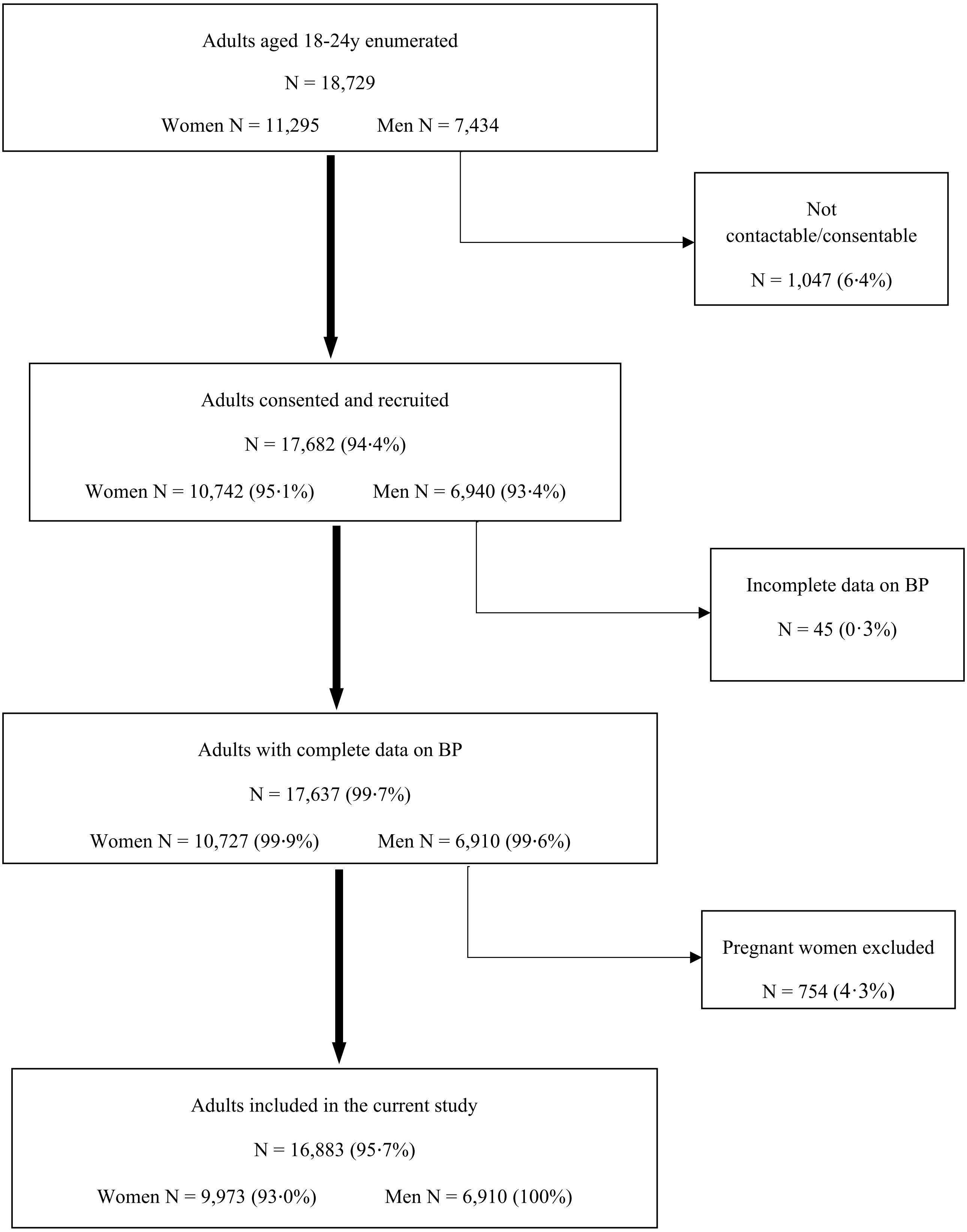

*Denominators for proportions are the total number in the tier above (for gender specific proportions, N for relevant gender in tier above

Supplementary information on data management:

Survey data were collected onto electronic tablets using the SurveyCTO (Cambridge, USA) platform and uploaded to a secure server at the end of each day, using a secure the Biomedical Research and Training Institute (BRTI) internet connection. Data were downloaded and stored in a password-protected database utilising Microsoft Access to execute quality control queries, with access limited to defined study personnel. Data were analysed using Stata v17.0 (StatCorp, College Station, TX, USA).

Supplementary Figure 2: Distribution of population systolic and diastolic BP

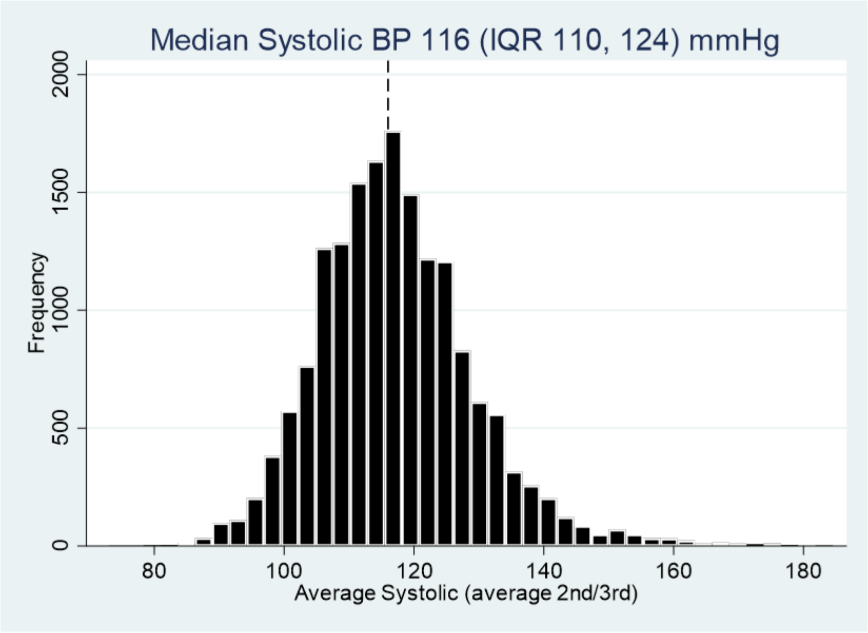

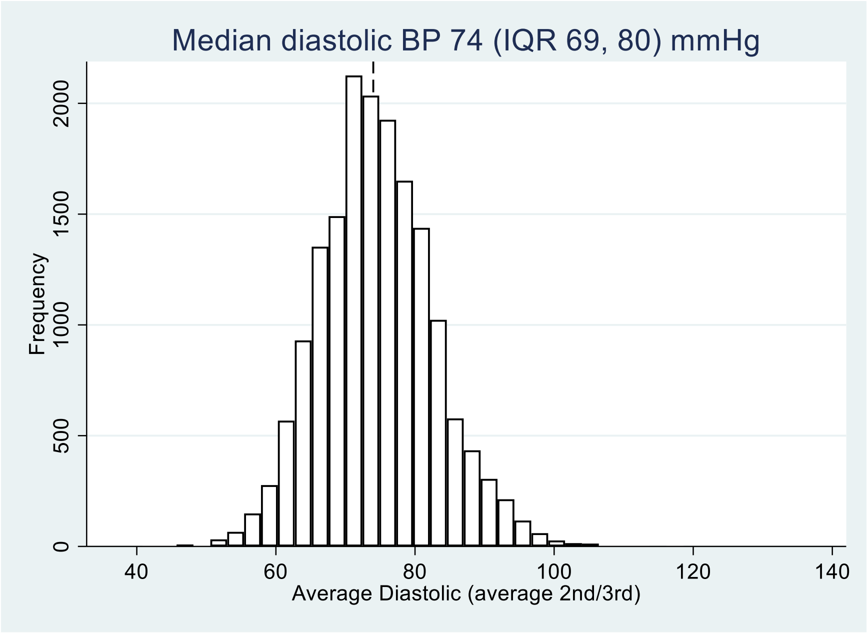

Supplementary Figure 3: Systolic hypertension (irrespective of diastolic BP) by BMI category

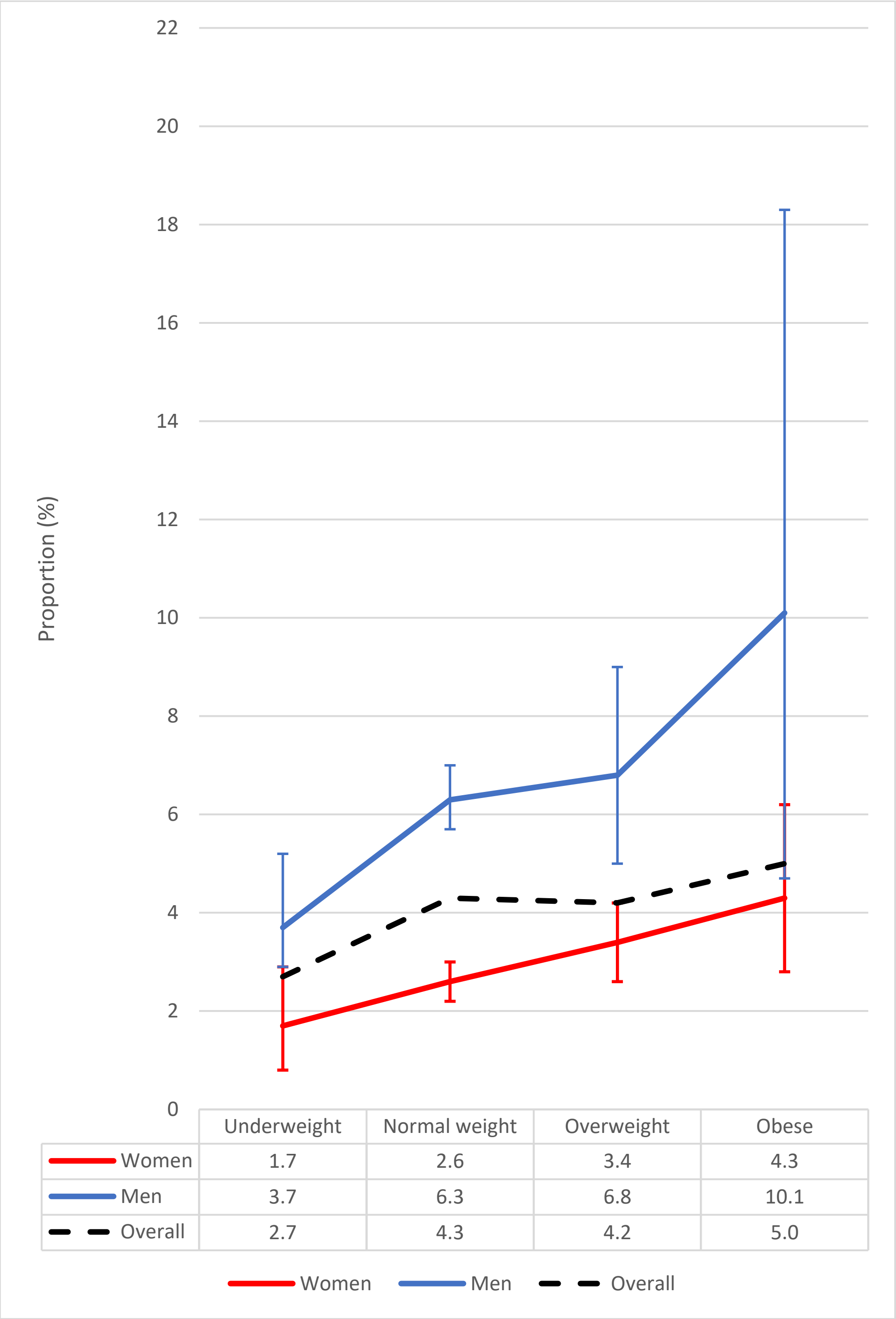

Supplementary Figure 4: Prevalence of obesity with age

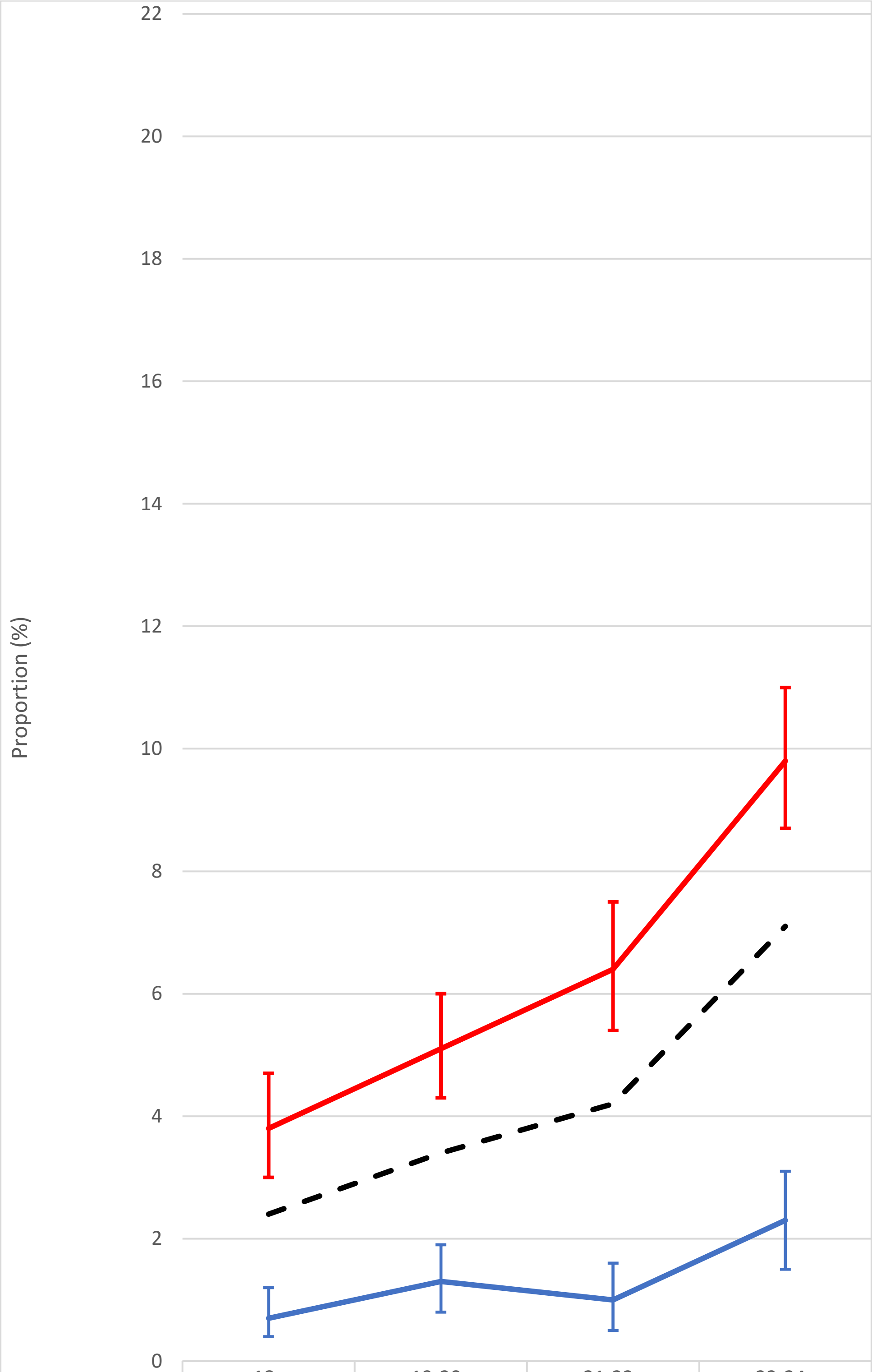

	18y	19-20y	21-22y	23-24y
Women	3.8	5.1	6.4	9.8
Men	0.7	1.3	1.0	2.3
Overall	2.4	3.4	4.2	7.1

Supplementary Table 1: Median BP measures at first, second and third reading

**BP measurements**<br>N=16,883	**Median systolic BP (IQR)**<br>**mmHg**	**Median diastolic BP (IQR)**<br>**mmHg**
1st measurement	118 (110, 126)	75 (69, 81)
2nd measurement	116 (109, 125)	74 (69, 80)
3rd measurement	116 (109, 124)	74 (68, 80)

Supplementary Table 2: Factors associated with high-normal BP

	High-normal (Systolic 130-139/ Diastolic 85-89 mmHg) (2,064/15,629)[1]	Crude odds ratio[2] (95% Confidence interval, LRT[4] p-value)	Multivariable odds ratio[3] (95% Confidence interval, LRT[4] p-value)
**Sex**		p<0.001	p<0.001
Female	1033 (11.1%)	1	1
Male	1031 (16.4%)	1.63 (1.48, 1.79)	1.70 (1.54, 1.89)
**Age category (years)**		p<0.001	p<0.001
18	426 (11.0%)	1	1
19-20	521 (11.6%)	1.04 (0.91, 1.20)	1.01 (0.88, 1.17)
21-22	515 (14.2%)	1.33 (1.16, 1.53)	1.28 (1.11, 1.49)
23-24	602 (16.6%)	1.58 (1.38, 1.81)	1.52 (1.31, 1.77)
**Highest education attained**		p=0.005	p=0.16
Primary or below	379 (13.3%)	1	1
Secondary form 4	1210 (12.7%)	0.97 (0.85, 1.10)	0.90 (0.80, 1.03)
Secondary form 6	270 (13.4%)	1.05 (0.89, 1.25)	0.93 (0.78, 1.11)
Higher education (above Form 6)	205 (16.6%)	1.32 (1.10, 1.60)	1.08 (0.87, 1.32)
**Occupation**		p<0.001	p=0.25
In education	576 (12.7%)	1	1
Employed/own business	101 (13.9%)	1.09 (0.87, 1.37)	0.93 (0.74, 1.18)
Work in informal sector	435 (15.5%)	1.25 (1.09, 1.43)	1.14 (0.98, 1.33)
None of the above	952 (12.6%)	0.97 (0.87, 1.09)	1.06 (0.93, 1.20)
**Socio-economic quintile**		p=0.17	p=0.48
Lowest quintile (least affluent)	468 (13.6%)	1	1
Second lowest quintile	367 (13.3%)	1.02 (0.88, 1.19)	0.98 (0.85, 1.14)
Middle quintile	403 (12.8%)	1.01 (0.87, 1.18)	0.92 (0.79, 1.07)
Second highest quintile	382 (12.3%)	0.98 (0.84, 1.15)	0.89 (0.76, 1.03)
Highest quintile (most affluent)	444 (14.1%)	1.16 (1.00, 1.36)	0.98 (0.84, 1.14)
**Physical activity (MET)[5]**		p=0.02	p=0.28
Low	662 (12.0)	1	1
Moderate	699 (13.6)	1.14 (1.02, 1.28)	1.09 (0.97, 1.22)
High	650 (13.6)	1.16 (1.03, 1.31)	1.02 (0.90, 1.14)
Don't know/Missing	*53 (32.5)*	-	-
**Alcohol/risk of problem drinking**		p=0.26	p=0.11
Never drink/low risk alcohol	1,960 (13.2)	1	1
Increased risk - possible dependence (AUDIT score ≥8)[6]	102 (14.0)	1.13 (0.91, 1.41)	0.83 (0.66, 1.05)
Don't know/Missing	*2 (15.4)*	-	-
**Smoke**		P=0.002	P=0.74
Never smoker	1908 (13.0%)	1	1
Ever smoker	156 (16.2%)	1.35 (1.13, 1.61)	0.97 (0.80, 1.17)
**Shona symptom questionnaire**		p=0.10	p=0.45
Low risk of common mental disorder	1,939 (13.3%)	1	1
Risk of common mental disorder (SSQ score ≥8)[8]	125 (11.7%)	0.85 (0.70, 1.03)	0.93 (0.76, 1.13)
**BMI category**		p<0.001	p<0.001

Underweight	154 (10.2%)	0.80 (0.67, 0.95)	0.77 (0.64, 0.92)
Normal weight	1422 (12.9%)	1	1
Overweight	382 (15.5%)	1.24 (1.10, 1.41)	1.35 (1.19, 1.54)
Obese	106 (16.9%)	1.34 (1.08, 1.67)	1.55 (1.24, 1.94)
**HIV status**		p=0.02	p=0.02
HIV-negative	1,936 (13.4)	1	1
HIV-positive	120 (10.9)	0.80 (0.66, 0.97)	0.80 (0.66, 0.98)
Missing/confirmatory results unavailable	*8 (17.4)*	-	-

1. Excludes participants with hypertension (n=1,254); 2. Crude OR adjusted a priori for cluster; 3. Separate models for each risk factor adjusted for sex, age, education, occupation, physical activity, smoking, BMI and HIV status; 4. Likelihood Ratio Test; 5. The International Physical Activity Questionnaire was used to ascertain levels of physical activity, expressed as multiples of the resting metabolic rate (MET) in MET minutes; 6. The Alcohol Use Disorders Identification Test (AUDIT), threshold of 8 on AUDIT score: low risk <8 and high risk ≥8; .7. Standard SSQ thresholds of ≥8 to indicate risk of common mental disorders was used vs <8 for low risk.
